# Supplementary material for: Molecular architecture and platelet-activating properties of small immune complexes assembled on heparin and platelet factor 4
Source: Commun Biol. 2024 Mar 11;7:308. doi: 10.1038/s42003-024-05982-4 (PMC10928113; doi:10.1038/s42003-024-05982-4)
Supplement: Supplementary file 1 — Supplementary Information [file 42003_2024_5982_MOESM1_ESM.pdf]

Supplementary Material for

Molecular architecture and platelet-activating properties of small immune complexes assembled on heparin and platelet factor4

Yang Yang, Yi Du, Daniil Ivanov, Chendi Niu, Rumi Clare, James W. Smith, Ishac Nazy and Igor A. Kaltashov

Table of Contents:

Figure S1	2
Figure S2	3
Table S1	4-5
Figure S3	6
Figure S4	7
Figure S5	8
Figure S6	9
Figure S7	10
References	11

Figure S1. Mass distribution of SCs obtained by applying the SEC calibration to the chromatographic peak within the 9.5-12 min elution window (the original unprocessed chromatogram is shown in **Figure 2** of the paper).

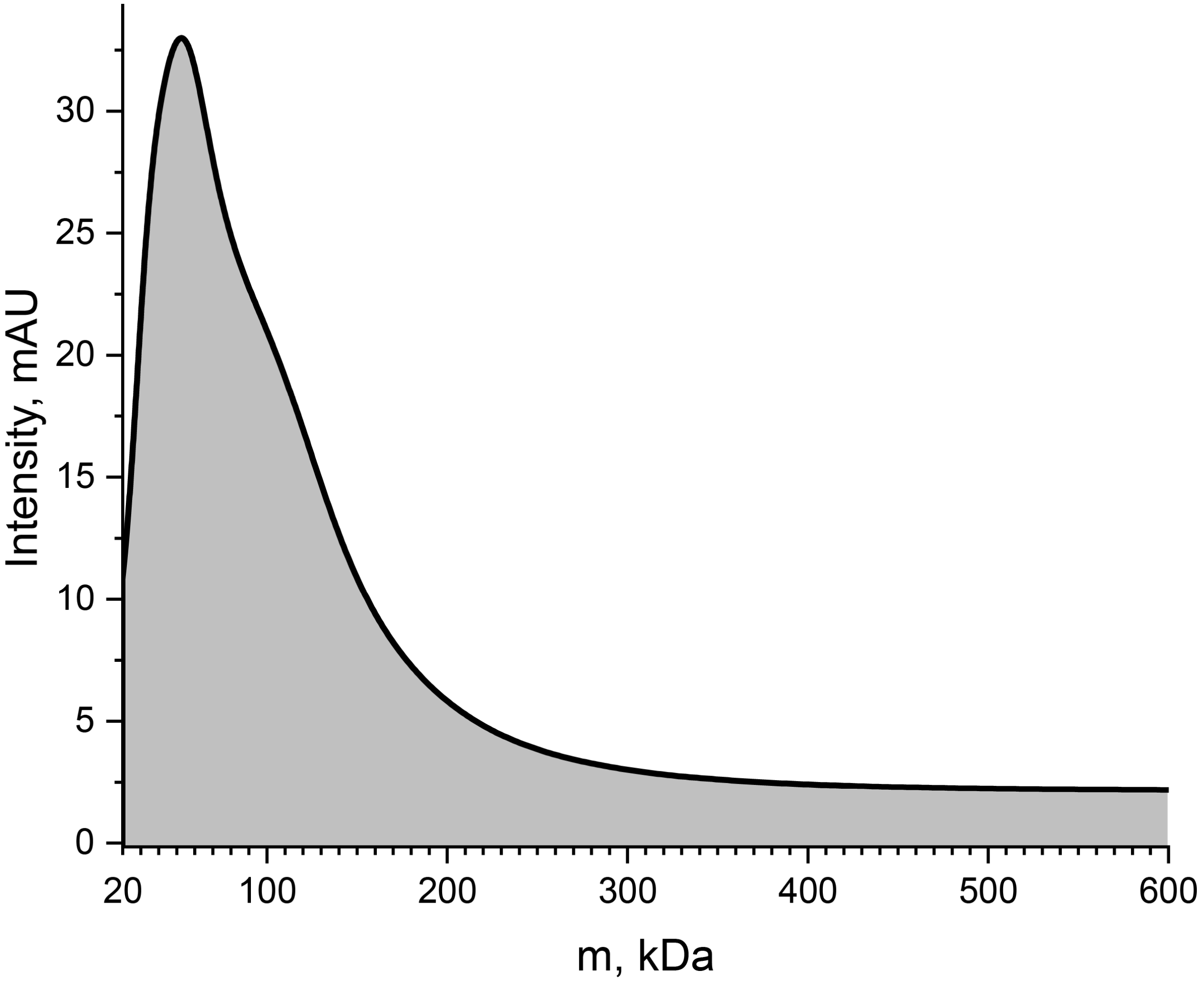

Figure S2. Native MS of UFH (0.25 mg/mL in 150 mM ammonium acetate) and mass assignment for representative ionic populations based on limited charge reduction measurements.

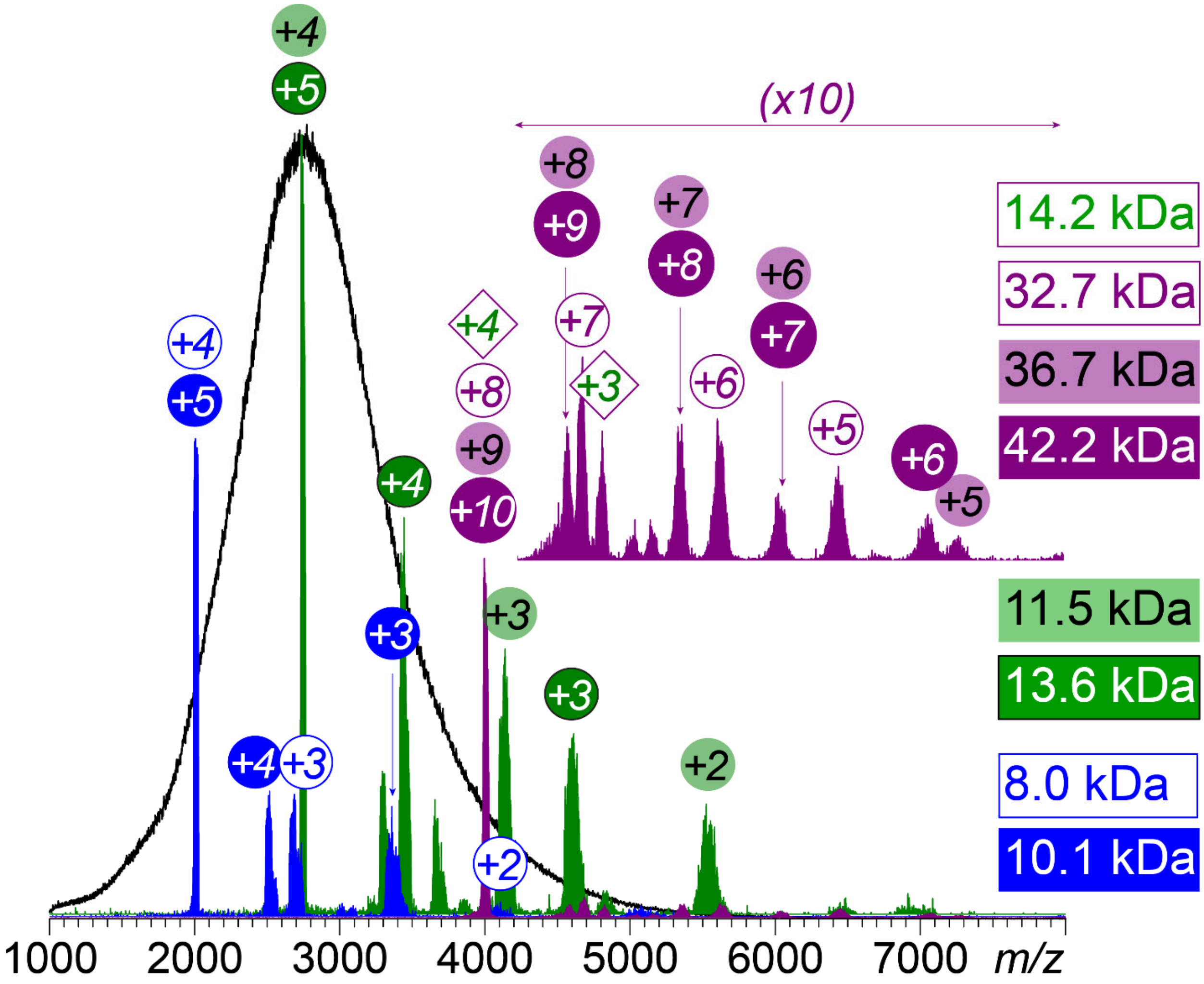

Table S1. Expected mass ranges for UFH/PF4/KKO complexes with different stoichiometries.

Experimentally Observed Small Complexes (SCs)

composition/stoichiometry	mass, kDa
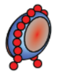	48.5 (44 - 50)
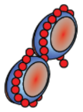	82-88
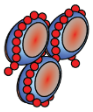	122-129

Putative Immune Complexes Built Upon the Observed SCs

composition/stoichiometry	expected mass, kDa
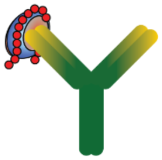	~ 200
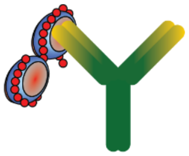	233-240
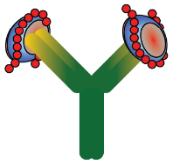	~ 249
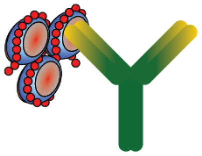	273-281
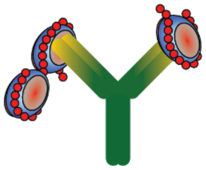	282-286

Table S1 (cont'd). Expected mass ranges for UFH/PF4/KKO complexes with different stoichiometries.

Putative Immune Complexes Built Upon the Observed SCs

composition/stoichiometry	expected mass, kDa
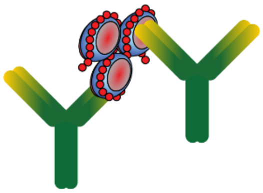	425-432
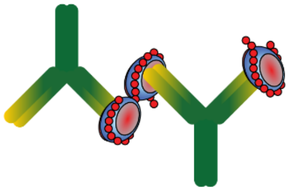	433-440
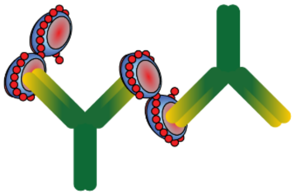	468-479
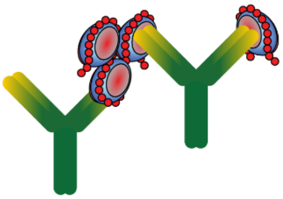	474-480
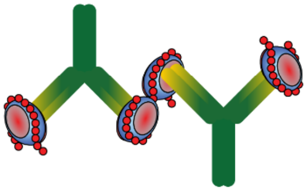	482-488
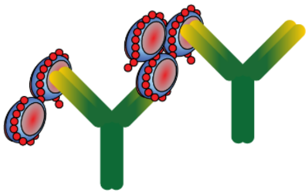	508-520
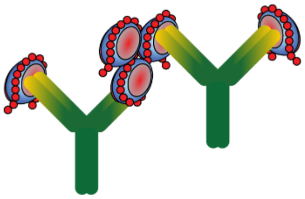	522-529

Figure S3. MD simulations of the dp100·$(PF4)_3$ complex formation using three different starting positions (shown in three different colors in the graph). The gyration radius collapse within the initial 100 ns in all three cases leads to formation of compact structures that remain stable during extended time intervals due to the tight wrapping of the polyanionic heparin chain along the equatorial positively charged regions of all three PF4 tetramers. The snapshots on the right-hand side illustrate the evolution of the extended structure towards the compact one (the corresponding trajectory is shown with the black trace on the R_g vs. time graph).

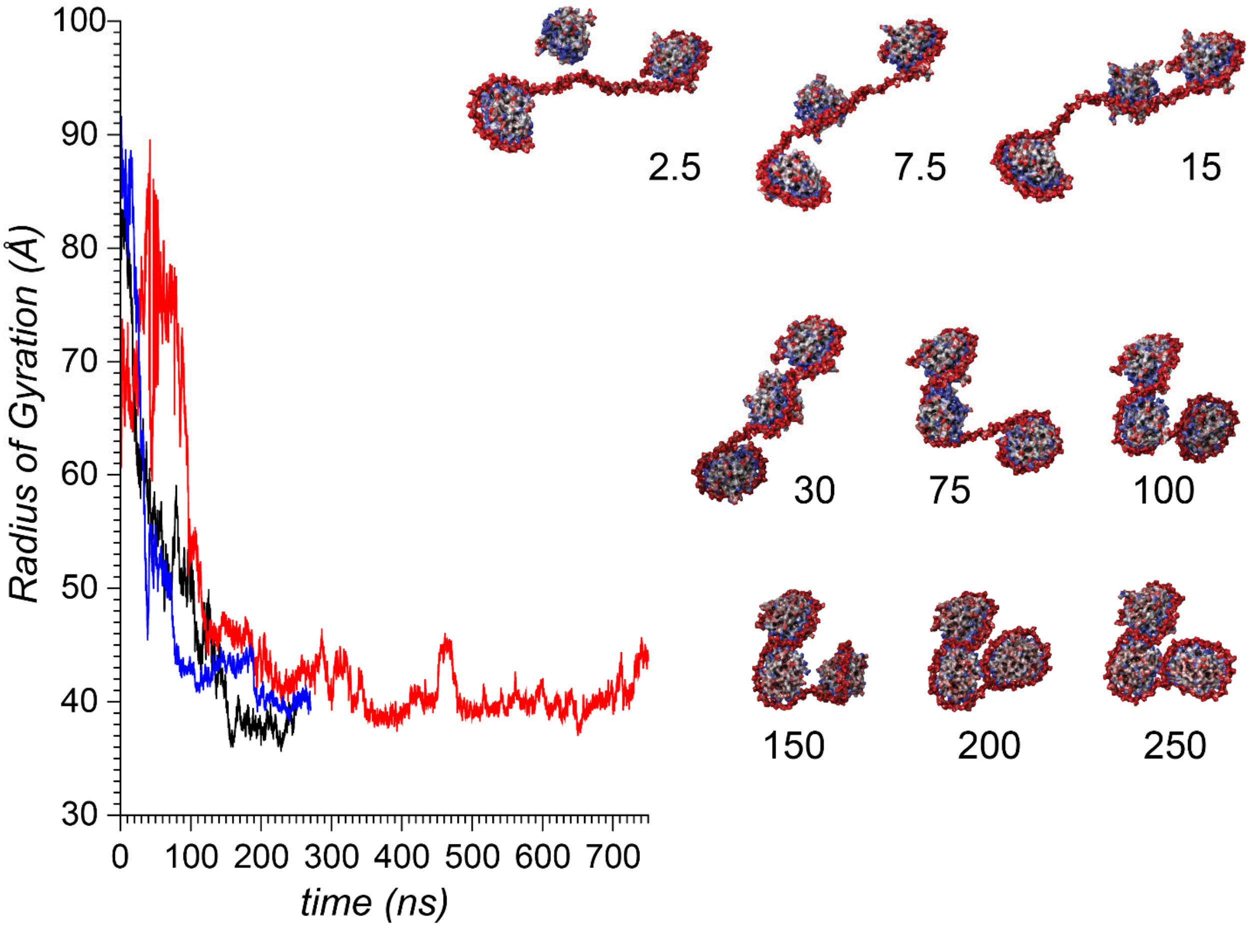

Figure S4. SRA data showing the platelet activation by KKO incubated with a UFH/PF4 mixture without fractionation of the immune complexes (blue bars), and after isolating fractions containing two and one KKO molecules per immune complex (fractions A and B represented with the violet and purple bars, respectively); platelet activation is shown as the percent release of ^{14}C-serotonin. The left and right bars in each group represent the data acquired with recombinant and blood-derived forms of PF4, respectively. The control measurements are shown with the black and gray bars (KKO alone and KKO/heparin mixture, respectively). Platelets used in the SRA assay were collected from Donor 2.

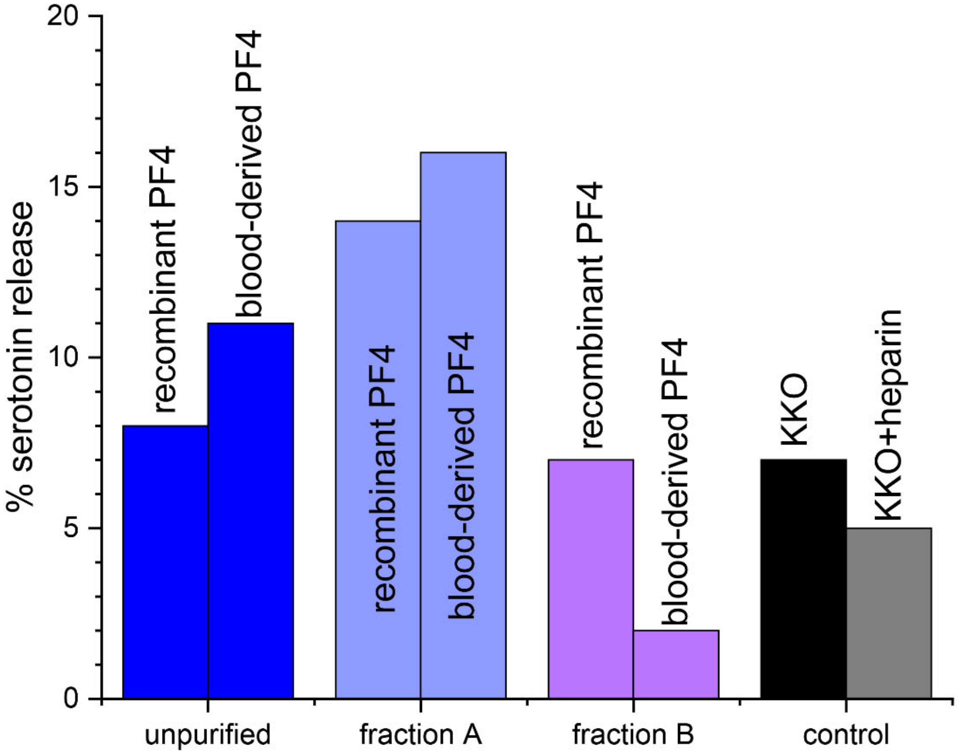

Figure S5. A structure of the dp100·$(PF4)_3$·($Fab^{KKO})_2$ complex produced by forced docking of the two Fab segments to their epitopes on the PF4 surface in *cis*-conformation. The paratope regions of the two Fab segments were forced to make contacts with the epitope regions on the surface of PF4 tetramers to make each antigen/antibody structure consistent with the crystal structure of the fondaparinux/PF4/Fab^{KKO} complex (PDB: **4R9Y**). The initial docking was followed by energy minimization. The imposition of the restraints meant to force both Fab segments to dock to the dp100·$(PF4)_3$ complex in the "cis" fashion results in a very tight fit in the final structure and a significant entropic penalty, making formation of such a complex highly improbable.

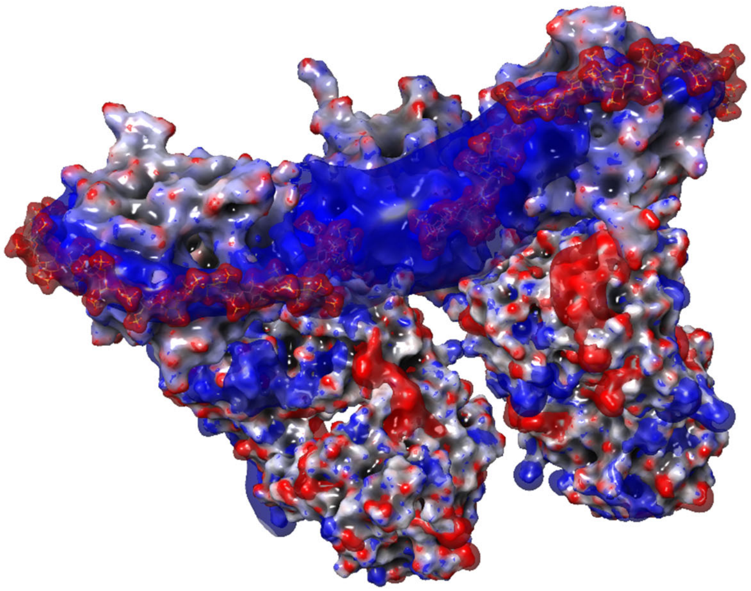

Figure S6. Native MS of ULCs generated in the UFH/PF4 mixture (0.67 mg/mL and 1.6 mg/mL, respectively). The unresolved ionic signal in the mass spectrum (*m/z* range 25,000-45,000) was used to estimate the average ULC mass (3.0±1.5 MDa) following the approach described elsewhere.[1]

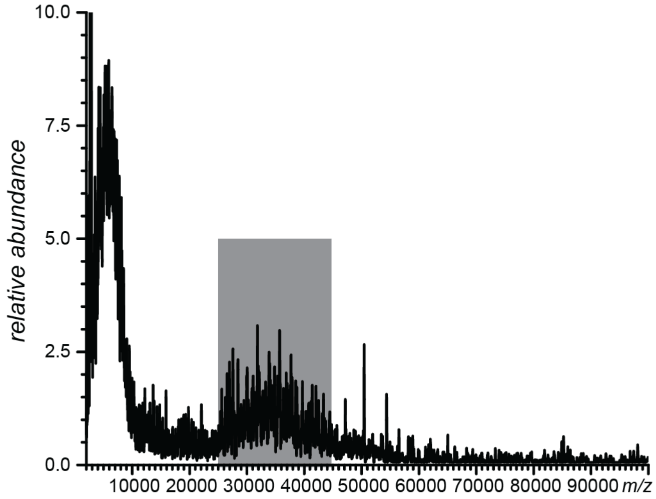

Briefly, the mass estimate of ULCs is made using the empirical relationship between the average ionic charge ($Z_{av.}$) and the solvent-exposed surface area of the corresponding species in solution (S):

$$Z_{av.} = AS^{\alpha}, \quad (1)$$

where A is a constant and the empirical value of α is 0.69±0.02.[2] Using a quasi-spherical approximation for the particle,[1] the mass (*m*) of the ULC particle can be expressed using its average radius of gyration *R* and effective density *ρ*:

$$m = \frac{4\pi}{3}R^3\rho \quad (2)$$

The surface area of the particle can then be expressed as:

$$S = 4\pi R^2 = (\frac{6\sqrt{\pi}m}{\rho})^{2/3}, \quad (3)$$

and substituting (3) in (1) allows the average ionic charge to be expressed as:

$$Z_{av.} = A \cdot \left(\frac{6\sqrt{\pi}}{\rho}\right)^{\frac{2\alpha}{3}} \cdot (m)^{\frac{2\alpha}{3}} = K \cdot (m)^{\frac{2\alpha}{3}} \quad (4)$$

This allows the relationship of the particle mass *m* and the *m/z* value of the corresponding ion to expressed as:

$$m = C \cdot (\frac{m}{z})^{\frac{3}{3-2\alpha}} \quad (5)$$

Since in our approximation this *m* vs. *m/z* relationship should hold true for both ULC and SC particles, the mass of the former can be estimated as:

$$m_{ULC} = m_{SC}\left[\frac{(m/z)_{ULC}}{(m/z)_{SC}}\right]^{\frac{3}{3-2\alpha}}, \quad (6)$$

where the empirical value of α is 0.69±0.02,[2] and all other parameters on the right-hand side of the expression are measured experimentally, providing the following estimate the average ULC mass: m_{ULC} = 3.0±1.5 MDa.

Figure S7. The proposed mechanism of ULC assembly. The successful chain propagation requires that at every step of the assembly process there be a significant mismatch between the “vacant” heparin segment length and the circumference of the PF4 tetramer.

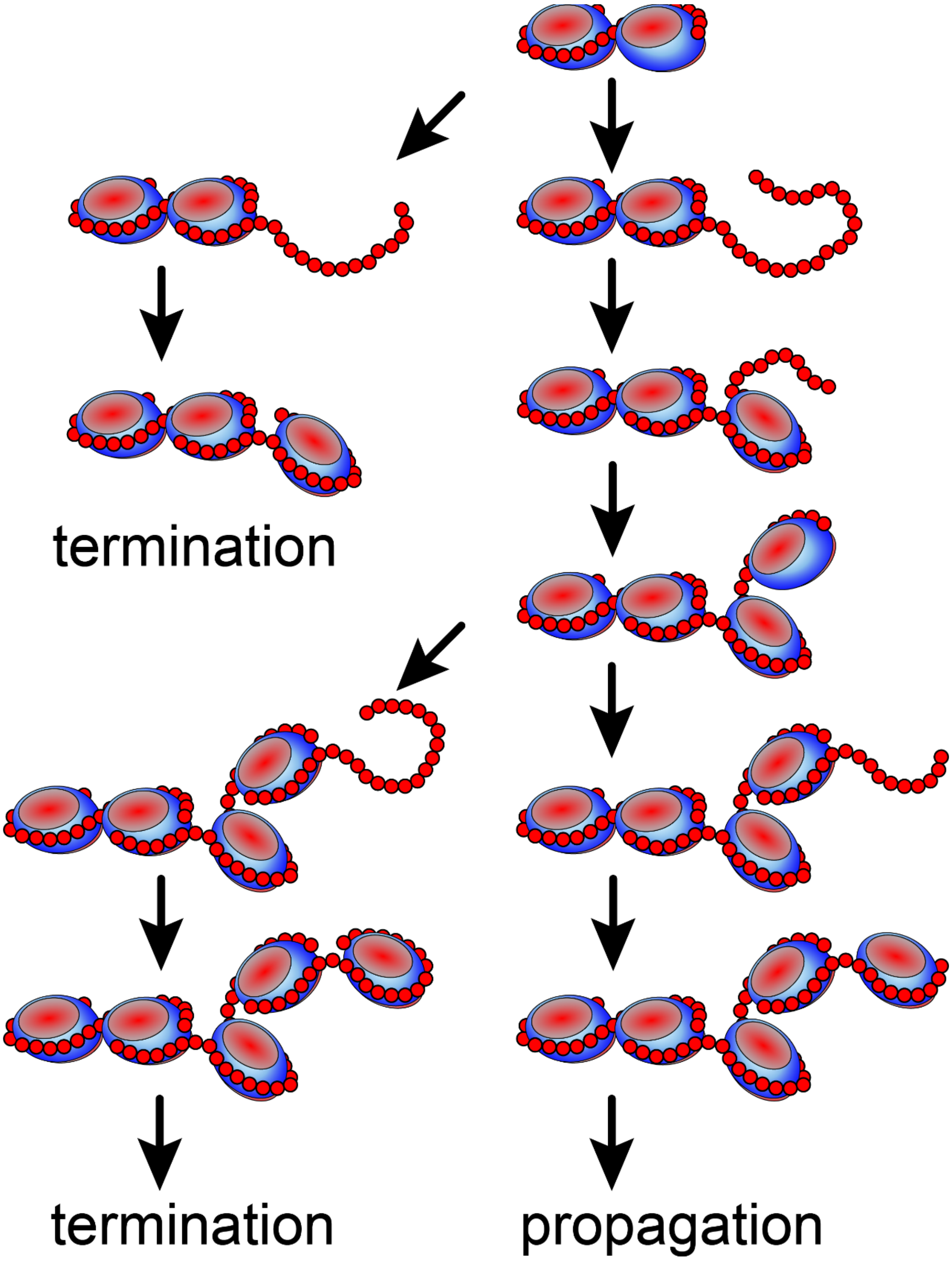

Supplementary References

1 Yang, Y., Ivanov, D. G. & Kaltashov, I. A. The challenge of structural heterogeneity in the native mass spectrometry studies of the SARS-CoV-2 spike protein interactions with its host cell-surface receptor. *Anal. Bioanal. Chem.* **413**, 7205-7214, doi:10.1007/s00216-021-03601-3 (2021).

2 Kaltashov, I. A. & Mohimen, A. Estimates of protein surface areas in solution by electrospray ionization mass spectrometry. *Anal. Chem.* **77**, 5370-5379 (2005).
